# Can we import quality tools? a feasibility study of European practice assessment in a country with less organised general practice

**DOI:** 10.1186/1472-6963-9-183

**Published:** 2009-10-11

**Authors:** Roy Remmen, Luc Seuntjens, Dominique Paulus, Dominique Pestiaux, Klaus Knops, Ann Van den Bruel

**Affiliations:** 1 Department of General Practice, Geriatrics and Interdisciplinary care, Faculty of Medicine, University of Antwerp, Antwerp, Belgium; 2 Belgian Health Care Knowledge Centre, Brussels, Belgium; 3 Department of General Practice, Université Catholique de Louvain, Brussels, Belgium; 4 Department of General Practice, Faculty of Medicine, Catholic University Leuven, Leuven, Belgium

## Abstract

**Background:**

Quality is on the agenda of European general practice (GP). European researchers have, in collaboration, developed tools to assess quality of GPs. In this feasibility study, we tested the European Practice Assessment (EPA) in a one-off project in Belgium, where general practice has a low level of GP organisation.

**Methods:**

A framework for feasibility analysis included describing the recruiting of participants, a brief telephone study survey among non-responders, organisational and logistic problems. Using field notes and focus groups, we studied the participants' opinions.

**Results:**

In this study, only 36 of 1000 invited practices agreed to participate. Co-ordination, administrative work, practice visits and organisational problems required several days per practice. The researchers further encountered technical problems, for instance when entering the data and uploading to the web-based server. In subsequent qualitative analysis using two focus groups, most participant GPs expressed a positive feeling after the EPA procedure. In the short period of follow-up, only a few GPs reported improvements after the visit. The participant GPs suggested that follow-up and coaching would probably facilitate the implementation of changes.

**Conclusion:**

This feasibility study shows that prior interest in EPA is low in the GP community. We encountered a number of logistic and organisational problems. It proved attractive to participants, but it can be augmented by coaching of participants in more than a one-off project to identify and achieve targets for quality improvement. In the absence of commitment of the government, a network of universities and one scientific organisation will offer EPA as a service to training practices.

## Background

Quality is on the agenda in European general practice [1]. Many countries have set up schemes to develop and assess quality. Quality development initiatives are performed at different levels. At the individual level, the GP improves his/her work for instance by up-grading personal certification and accreditation levels or simply to fulfil personal learning needs. The next level (the practice) takes into account the premises of the practice, practice organisation and the interaction between health care workers in the practice. At a higher level, local or regional groups of GPs organise projects to improve quality for instance through peer review groups. The central level relates mostly to initiatives by colleges for general practitioners or governments for, e.g. standard setting, guideline development and feedback on prescription and formal certification and accreditation procedures [2].

From 2002 to 2005, researchers collaborating internationally developed the European Practice Assessment (EPA) to assess the organisation of GP practices. The EPA tool was first tested in more than 270 practices in Europe [3]. The aim of EPA is to have an impact on the quality improvement on the premises of the practice and in practice organisation. It is an instrument to enable benchmarking practices for different indicators [4]. EPA analyses five domains i.e., 'infrastructure', 'people', 'information', 'financial management' and 'quality and safety'. Every domain is subdivided into so-called "dimensions". The dimension indicators were selected after literary reviews and consensus techniques within the European Association for Quality in General Practice/Family Medicine (EQuiP), a network organisation of the European branch of the World Association of Family Doctors (WONCA) [5]. The domain 'infrastructure' addresses aspects of the premises, accessibility of the practice and medical equipment. The working conditions, the training and education, the perspective of the patients and staff members are assessed in the domain 'people'. The domain 'information' includes aspects such as the protection of privacy, practice brochure, specialized information for physicians and other practice members. The domain 'quality and safety' assesses how the practice minimizes risks for patients and staff and how the practice handles patient complaints. Finally, the domain 'financial management' includes aspects on leadership and financial planning.

Currently, EPA is used in a comprehensive quality development program and used as a assessment tool in Germany and Switzerland [6]. In Germany systematic quality development at the practice level has been made obligatory for all medical doctors working in ambulatory care, and EPA is one of the instruments available in this country [7]. It is also being pilot tested in Romania, Denmark, Sweden, Qatar and Saudi Arabia. Derivates of EPA are used in the Netherlands where it is part of a three year accreditation program [8]. In Australia, comparable practice based accreditation is offered by two organisations [9].

The countries that use quality development strategies at practice level usually rely on a well-structured organisation of the primary care system [10]. The income of GPs is higher compared to the average in other European countries and practices are supplied with auxiliary staff [11,12]. The implementation of quality assessment procedures at practice level may be more difficult in countries with a less organised general practice (having no gate-keeper role, lower income, small practice size, majority of GPs working in sole practice without auxiliary personnel) because GPs in these countries do not have the resources for these extra tasks.

The first report of EPA showed that it proved possible to develop a European set of indicators for assessing the quality of practice management, despite the differences in health care systems and cultures in the six different countries [13]. However at that time, when testing EPA across Europe, the authors used highly selected practices and therefore generalization of their findings is difficult and feasibility per country was not assessed. In countries with less privileged general practice one could pose the question, is the ground ready for using improvement strategies at practice level?

This feasibility study was performed in Belgium and focuses on the acceptability and applicability of the EPA tool in general practice where there is no quality improvement strategy at practice level. This paper reports measures of feasibility, including acceptability among GPs, reasons for non-responding, organisational problems and also the opinions of the participating GPs. The professional GP organisations (one Dutch speaking and one French speaking) and two academic departments organised and carried out this pilot that was requested and funded by the Federal Government.

## Context: General practice in Belgium is low profile

Belgium is a small tri-lingual country with 10.600.000 inhabitants situated between France, the Netherlands and Germany. It is a high income country and the health expenditure is in the average European range, with total health expenditure as a percentage of GDP of 10.2 percent [14]. GPs have no gate-keeping role[12] and their income is low compared to countries with better established general practice such as the UK and the Netherlands [11,15].

General practitioners generally work in a fee-for-service system, only a small proportion opts for a system based on capitation and there is out-of-pocket payment. The average size of practices is around 1100 patients per GP which is small by European standards. The percentage of solo-working GPs is rather high compared to European figures [16]. The income per GPs is in the average range in Europe [11].

The majority of GPs work in solo practice, and generally do not have auxiliary personnel. The Federal Government proposed funding for practices (duo and group practices) to attract auxiliary personnel from late 2008 onwards. Accreditation is only at individual GP level, i.e. attendance of post gradual teaching (20 hrs per year) and attendance of Local Quality Groups (2 sessions per year). Feedback about prescriptions is offered to all GPs by the central government. For quality development, the practice level has not been addressed at this stage [17]. There are two professional organisations both showing interest in quality improvement [18,19].

## Methods

The trial at stakeis part of a study commissioned by the Belgian Health Care Centre (KCE). Since the KCE is a public service and the trialis not interventional the approval of an ethical commission was not legally required according to Belgium law.

A framework for analysis of feasibility encompasses the description of recruitment of participants, a brief telephone survey study among non-responders, organisational and logistic problems. Using focus groups, we studied the opinions of participants.

### Recruitment of participating GPs

The sampling procedure aimed at recruiting 40 GPs working in different settings. We set out to find 20 GPs working in a single-handed practice, 12 in duo practice and 8 in group practice. A random sample of 500 GPs was first contacted by post emphasising that the EPA procedure was offered free of charge and was organised by the participating universities and the scientific organisations. The letter of invitation was signed by academics and the senior members of professional organisations, asking them to participate in the EPA study. Given the low response rate, the researchers had to send 500 additional letters to a second random sample of GPs. Moreover, telephone calls were made to a sample of 30 non-responders to inquire as to the reason for non-participation.

### The instrument

The research team used the latest English version of the EPA tool, which differed considerably from the previous version reported in 2005 [20]. They first translated the instrument including items, questionnaires, instruction forms and letters of correspondence into Dutch and French. Two bi-lingual authors checked the translations twice.

The practice visitors entered the data of the practices using the IT platform of EPA called Visotool^® ^which is currently hosted at the AQUA-institute for Applied Quality Improvement and Research in Health Care (Göttingen, Germany) [6]. The AQUA-institute offered training and continuous coaching throughout the project.

### Logistics

To support future manpower availability (sustainability for future projects) we decided to work with six practice visitors who made an average of 6 visits. All but one were GPs. The team organisation was supported by two co-ordinators and by auxiliary staff from the Department of General Practice of the University of Antwerp and they were trained during a one day training session.

After agreeing on a visitation date, two team members personally delivered, or sent by post with a telephone follow-up, all questionnaires and study material, and clarified any remaining questions. The analysis, interpretation and feedback of the findings took place on the day of the visit, on-the-spot during a team-meeting within the GP practice. The results were shown to the GP and the practice team, by use of a wireless internet connected laptop using the Visotool^®^. In the event of connection problems and for back-up reasons the feedback report was also downloaded as a PDF file. After the visit practices received written feedback reports.

### Qualitative evaluation of the process

A brief telephone survey was performed among 30 non-responders selectively drawn from the total list. Each GP was asked the main reason for non-participation.

A further qualitative analysis was performed in two phases. The visitors were requested to complete standardised field notes after a visit, addressing the following questions:

1. What were the practice and visitor's first impressions of this EPA visit?

2. Did the practice workers perceive the EPA visit as useful?

3. Did the practice workers have any idea or plans for quality development following this EPA visit?

The field notes were summarised in one file and summarised by one researcher (RR).

Four weeks after the visits, all GPs who participated in the EPA study were invited by the researchers to participate in two focus groups. After one email, 16 GPs all from different practices, agreed to attend the focus groups and they were equally divided over two of the countries languages. Each focus group lasted 90 minutes.

Three focussed questions were addressed:

1. How did the GPs experience the EPA evaluation?

2. Was this model of practice-evaluation useful for their practice?

3. Is this model of practice-evaluation useful and applicable for the quality evaluation of general practitioners in general?

Two focus groups were held (8 participants in each group, one in each language). The main aim was to get diversity of opinions. The discussions were led by a trained moderator with expertise in qualitative research and an external observer assisted who had not been involved in any of the practice visits.

The discussions were typed out verbatim. After the discussion, the trained moderators and the observers wrote up and agreed on the general perspectives of the three topics and they made a first report. Subsequently, the typed out reports were analysed by two authors (RR and DPest, who did not attend the focus groups). They went through the texts and identified relevant statements and wrote a preliminary draft. Subsequently these two drafts went into one final report which also included information from the first reports of the moderators and observers.

## Results

### The organisational process

#### Recruitment of GPs for the EPA visit

The first mailing to 500 GPs (250 each for both languages) resulted in 10 participants in Flanders and 6 in the Walloon region. A second mailing to 500 GPs yielded 17 more candidates in Flanders and 10 candidates in the Walloon region. Two additional French speaking GPs agreed to participate after personal invitation from the researchers. In Flanders it was possible to select 20 practices according to the proposed grid, in the Walloon region we had to select all 18 practices that were available.

Two initially enrolled solo practices decided to quit the project. The first practice declined for practical reasons. The second one refused to administer the patient questionnaires. According to this solo working GP, participation would require too much explanation to the patients, would be too time-consuming and interfered with the confidential doctor-patient relationship.

Finally, 36 practices participated (response rate 36%). These were not representative for the Belgian GP population because most of them were active as trainee supervisor, academic assistant or members of professional organisations. In table 1, key features of participating practices are detailed.

**Table 1:** Data of the 36 participating general practices, and national figures when existent.

	**Sample**	National (*)
Dutch speaking	19 (53%)	61
French speaking	17 (47%)	39
		

Mean number of GPs per practice	2,9 GPs/practice	No data, vast majority in solo practice

Mean age of GPs	43 years	49 years

% Male GPs	55%	81

Mean number of assistance staff in the practice	1,9 person/practice	No data

(*)National data available at

#### Telephone survey among non-responders

Thirty non-responders drawn from the first mailing were contacted by phone. In spite of receiving additional information about the EPA study, none of them was motivated to participate. The main reason (n = 16) was the overflow of patients at the time of the study (flu season). A substantial part (n = 6) no longer worked as GP or were not interested in the study (n = 5).

#### Preparations made by the facilitating team

All team members needed to be bi- or multilingual (Dutch/French and English), familiar with the EPA procedure and able to work with Visotool^®^. The co-ordinators and external visitors followed a course run by the AQUA-institute team on the content and development of EPA. External visitors had a further training session to improve communication skills which was of utmost importance for smooth communication with the practice workers. The non-doctor visitor performed equally well.

The provision of human resources initially planned was insufficient. The co-ordination, administrative work, preparation of the visit, visits to the practices, solving of practical problems and links with Germany required a few days per participating practice. Supplementary administrative support was necessary.

#### The practice visit

In the most efficient time schedule, a practice visit lasted 4 hours. Due to technical problems and delays in returning the completed questionnaires, half of the visits were spread over two days.

#### Technical support

During the visit and the team-meeting, the external visitors used Internet connection by UMTS technology to present the results directly from the Visotool^® ^website. Unfortunately, technical support was necessary during half of the visits, due to problems with logging into the UMTS network, the use of the portable computers or getting access to the Visotool^® ^website. In most cases, a Belgian researcher solved the problem but one third of the practice visits required technical support from the German helpdesk. Some GPs working in single-handed practices suggested feedback visits on Saturdays, which was not feasible because the German support was not available out-of-hours.

### Qualitative evaluation using field notes

15 field notes were collected. At the end of the visit, most participants showed a positive attitude towards EPA. They generally appreciated the visit as a peer review process to objectively evaluate the practice organisation. The topics of interest were mainly the emergency medications, complaint management, GPs' vaccination status follow-up (e.g. hepatitis A and B), patient information on practice organisational topics and fire-safety.

Many GPs appreciated the confidentiality of the procedure: personal contacts between co-ordinators and practices were rated highly useful. Several GPs expressed concerns on confidentiality for any other use than within this project: they stressed that they would never allow the access of their data to controlling agencies.

Some GPs found that the questionnaires contained items that were hard to understand. This sometimes caused doubt on the interpretation of these items for both the GPs and the practice visitors. GPs working in single-handed practice frequently noted that items on practice organisation were applicable for group practice only. Some items were not adapted to the local context or out-of-date (for example the use of videotapes for patient information). Some GPs found items missing, such as disinfectant procedures.

Some participants found the time-schedule and preparation time insufficient. Not all GPs were present to check their doctor's bag which is one of the sub-domains of EPA. Despite written information and frequent information sessions by telephone, some GPs did not have a clear idea of the procedures on the visit day. All GPs appreciated the feedback and reported that the presentation of that data was clear.

### Qualitative evaluation using focus groups

#### Experience of the GPs: enthusiasm but fear of external control

In general, GPs were enthusiastic about the transparency they experienced between colleagues in their practice and with the visitor. They felt supported by the findings of the report of their practice and were satisfied that new and interesting domains of their practice were highlighted. They especially valued receiving, for the first time, their patients' appreciation. EPA also triggered reflection among other colleagues within the practice.

'Everybody in the practice perceived EPA as extremely interesting; we are taking a closer look at domains where we scored sub-optimal. Can we change, and should we change? My colleagues were enthusiastic about these new dimensions' (Dutch speaking GP)

Participants suggested that peer review groups (mandatory in Belgium) could be useful to discuss results and to propose further improvement. All participants expected a more thorough appraisal with extra coaching, especially on organisational aspects of their practices. For instance, they felt that the report of the five domains, which in the software is visually represented as a pantograph, needed more detailed explanation.

'We may need a more thorough appraisal, so to speak, to achieve the ISO 2000' (Dutch speaking GP)

They opposed the idea of inspection, but favoured introspection. Many participants were afraid that the evaluation and validation would standardise all GP practices: some GPs called this phenomenon "normalisation". Some GPs also argued that the results could give a sense of guilt when not achieving the highest standards. Overall they felt that EPA did not entirely fit into their reality. Many elements of the questionnaires and checklist were seen as not relevant, sometimes uninteresting or even 'ridiculous'.

'..many items are not applicable in Belgian general practice' (several GPs)

'..it was too much about group practice, they did not apply to single-handed practices' (Dutch speaking GP)

#### Usefulness for the practice: difficult to design plans for improvement

The GPs appreciated the freedom to implement an optional change in their practice. Only some participants in one group reported a few formal plans for improvement after the EPA visit, but at the time the focus group reported no solutions or worked out plans. They agreed that follow-up and coaching would facilitate the implementation of changes. Simple practical arrangements are easy to implement (e.g. a thermometer in the fridge) but other items are more difficult to deal with without any external support and follow-up. Implementing change seemed easier when working in a group practice. It was argued that some improvements (for instance making up a yearly report) were not achievable for most Belgian single-handed practices. The lack of time and having other priorities were also major reasons for not implementing changes.

'If it became a sort of routine as a typical practice visit, such as other existing formulas, that would be different, then you could effectively say: oh yes, that's not a bad idea, I could do that'

(Dutch speaking GP)

'We don't normally look at it from the point of view of the practice staff. We always think for them. But from the results of this study we realise they hold different views.' (French speaking GP)

The GPs were generally interested in patients' opinions of the practice. They had observed that their patients collaborated easily with the survey. In some cases, there were problems with practical arrangements for data collection, for instance logistics in the waiting rooms.

Some parts of the questionnaires were difficult to understand. Some argued they could be shorter and simpler. Furthermore, the importance of the items varied. For example, the item about the practice printer seemed less relevant but the access for disabled people was seen as more important. Also, the lack of items about the home visits was perceived as a drawback in Belgium, where about one third (check) of the contacts with patients occurred in the home.

The validity of the items is an important issue to convince the GPs that the tool is relevant for the profession. They also needed reassurance that the items in the questionnaires and in the checklist reflected the quality of care at patient level.

'They must make all items valid in our setting; especially if they want to be sure they can be used on a larger scale'

(French speaking GP)

The role of the visitor is very important. Some GPs argued that they did not receive the written evaluation report on time. Some GPs did not fully understand the report and they needed more coaching after the practice visit. This was especially argued by GPs working in a group practice.

## Discussion

We report a feasibility study of a European tool in Belgium, a small country in Europe. It was hard to attract a sufficient number of GPs indicating low prior motivation of the GP community. Furthermore, we encountered many problems during our project. Participating GPs were enthusiastic about EPA in their practice.

The problems we identified were not reported in the first study on EPA in 2005, probably because the participants in that study were a different subset of the GP population. Furthermore, our scope was different; we sought to find barriers and facilitators for future larger scale implementation. GPs in Belgium work in unfavourable conditions compared to other countries in Europe in which EPA-like instruments are currently in use. Our findings are important to countries which begin to show attention to quality issues in relation to the practice setting. France for example, where GPs work in similar conditions, recently adopted a federal law aiming to start implementing quality approaches at the practice level[21]

The researchers underestimated the investment in time and personnel. The co-ordination, administrative work, practice visits and organisational problems required several days per practice. Initially we estimated a full time equivalent GP researcher for three months to work on the project and this turned out to be a substantial underestimation. We also needed to employ extra secretary staff from our department. Also, additional technical support was necessary in half of the practice visits, due to problems visitors encountered with the internet connection when entering the data and uploading to the web-based server in Germany.

In the qualitative analysis, most participating GPs expressed a positive feeling after the EPA procedure, for them it was a very worthwhile effort. They appreciated the focus on their practice and the feedback that highlighted new domains (in particular the patient and staff feedback). They did not report on the technical problems during the visits probably because the visitors managed most problems without disturbance. Based on these findings we conclude that there is a niche for EPA in Belgium for this selected sub-population.

Our participants drew attention to a number of undesired details of going through the EPA procedure, which may hamper broad acceptance among the GP community.

Firstly, GPs in solo practice especially, felt that EPA did not entirely fit the reality of general practice in this country. In particular, GPs working in a single-handed practice (common in Belgium) found that several items were orientated towards the organisation of group practices. However, in other European countries like Germany, Switzerland and the Netherlands, many GPs also work solo and apparently do apply the EPA tool successfully. Their practices however, do employ auxiliary personnel. Moreover, in these countries medical insurers show interest, as shown by reimbursed schemes (Australia, UK and the Netherlands) or formal regulation (Germany) to address the quality at the practice level.

Secondly, a major finding was that GPs were concerned about the confidentiality of the data. They feared the potential use by the authorities, i.e. the summary use of the data. They stressed that data obtained from EPA should be handled with great care and preferably by the profession itself. They suggested that the implementation should be initiated on a voluntary basis; the anonymous data could allow a benchmarking of their practice with other practices. Obviously, when looking at general practice as a business, one needs adequate data input for management as is the case in Australia. In Australia a well accepted third party offers data to practices to allow them to implement better practice management [22]. Although nearly all practices in Belgium are privately owned by the GPs, this business concept needs more time before it can be introduced.

Thirdly, in this project EPA was a one-off project without any subsequent engagements. Only a few practices did indeed report implementation of changes in the focus groups. The quality circle, which addresses formative elements (i.e. measuring, improving, measuring), is essential for real improvement to occur [2]. The participants argued that only one visit, as performed in this pilot, is not enough to foster quality development initiatives in the practice. Participants spontaneously suggested the need for further coaching as in the Australian programs and in practice visits in the Netherlands [8,23]. To date, only limited data exists on the GPs behavioural change while using the EPA tool [24]. Maybe significant quality improvement could be achieved if the EPA tool was embedded in a broader concept of practice accreditation, as in the Netherlands and Australia for instance [8,25].

Increasing evidence shows that local and cultural factors need to be taken into account when introducing quality tools in a particular health system. Marshal examined the transfer of individual quality indicators for the use in quality development and concluded that in the view of experts, quality indicators could be interchanged across health systems [26]. Looking more closely we find that, although apparently based on the same evidence, the specific content of guidelines differs across countries. Therefore transfer of guidelines along with its indicators might not be so straight forward. For instance, Matthys et all concluded that choice of evidence and the interpretation for clinical practice differs for guidelines concerning one clinical condition [27]. In this study we tested an instrument that was built up of more than sixty indicators. As we show, the applicability of actually using these indicators is also hampered by the culture and constraints of a particular country.

This study highlights the gap between the availability of well-designed quality tools and the actual barriers in a country that does not have the professional culture or the organisational culture to implement it. Based on our experience we suggest that, apart from legislative initiatives as exist in Germany, there are major points to consider before importing EPA or other quality initiatives.

• There must be a basis of professional culture on quality improvement. In our study we found a lack of interest among the vast majority of GPs for quality initiatives. In contrast, we know that countries like Australia and the Netherlands, GPs show a professional vision and commitment [23]. To adjust the culture from direct clinical care towards a systematic quality development culture remains a challenge for the future.

• The second ingredient for success is the design of a quality framework that specifies among others the stakeholders, quality instruments, consequences of the measures, potential incentives, and confidentiality [25]. This framework should be supported and implemented by the professional organisations and health authorities.

• Pilot studies, such as reported here, are an unavoidable step before implementing any quality tool. The tools need to be adapted to the local context to avoid discomfort among participants when facing items not applicable to them. For international comparison one can choose not to change the instrument but rather explain to end users why some items might be less applicable to them.

• Finally, human and financial resources must be carefully estimated before launching any wide-scale initiative. In Australia, the budget devoted to the quality in GP is estimated around 5 euro per capita per year [11,28]. EPA in Germany costs about 1800 euro per year per GP [7]. For a sustainable project, the cost of the quality procedure itself has to be added to incentives for GPs who take part in quality improvement initiatives. For EPA to run on a wider scale in Belgium, we calculated the procedural costs at approximately 1000 euro per practice per year (one Euro per capita approximately), so this would mean about 3000 euro for a three year project (see also table 2). Offering EPA-like tools to GPs is a complex task. Its large-scale implementation requires a significant facilitating and organisational structure. The price for an EPA-like project can be assessed. Assuming that handbooks and procedures are adapted to a certain countries context, the hypothesis is that in the first year practices are enrolled and collect data. During the second and third year, tutors coach the practices (approximately 2 days of face to face contacts with the practice). For manpower, the price could be estimated around 600 euro per practice per year. Moreover, overhead, location, IT infrastructure and data engineering need to be included in the budget. As for Belgium, the costs for the entire project may be approximately 1000 euro per year per practice. Assuming that one GP serves about 1000 inhabitants, this would be 1 euro per patient per practice per year which is slightly more than 0,05 percent of the total health expenditure per capita.

**Table 2:** Overview for a sustainable project.

	**Role**	**Full time equivalents**
First year, 100 practices enrol	GP coordinator	0,5
	administrative support	0,5
	Tutor	1
		
Second year 100 more practices enrol	GP coordinator	0,5
	administrative support	0,5
	Tutor	1,5
		
Third year, a steady state, 300 practices are enrolled	GP coordinator	0,5
	administrative support	0,5
	Tutor	2

The practices are enrolled during the first year and are coached in the following two years. The manpower needed to adapt the instruments, to set up the IT platforms and data handling is not considered in this scheme.

The result of this study prompt the GP professional associations, academic departments and the government in Belgium to rethink the approach towards quality in general practice. In the European perspective, can they afford to lag behind? One year after locally marketing the results of our pilot, the Federal Government has not responded with financial incentives for the EPA project to go ahead, perhaps because there is no visible return of investment yet. As a consequence, Belgium remains focussed on traditional quality improvement initiatives like attendance of post graduate teaching, Local Quality Groups, and feedback on prescriptions. Although at considerable cost, none of these showed any improvement of quality at the practice level [17].

To carry on with the EPA project in Belgium, a network of universities and one scientific organisation will offer EPA as a service to training practices and 30 practices participate.

## Conclusion

This feasibility study shows that prior interest in EPA is low in the GP community. We encountered a number of logistic and organisational problems. It proved attractive to participants, but it can be augmented by coaching of participants in more than a one-off project to identify and achieve targets for quality improvement. In the absence of commitment of the government, a network of universities and one scientific organisation will offer EPA as a service to training practices.

## Competing interests

The authors declare that they have no competing interests.

## Authors' contributions

All authors helped with the design of the study. RR lead the project. DP and AVDB supervised the project on behalf of the Belgian Health Care Knowledge Centre. LS especially helped in the initial phase of the project. DPest, in coolaboration with RR performed analysis of the focus groups. RR wrote final versions and all authors helped work on the final text. All authors read and approved the final manuscript.

## Pre-publication history

The pre-publication history for this paper can be accessed here:



## References

[B1] Baker R, Wensing M, Gibis B, Saltman R, Rico A, Boerma W (2006). Improving the quality and the performance of primary care. Primary care in the driver's seat?.

[B2] Grol R (1995). Kwaliteitsbevordering voor en door huisartsen.

[B3] Engels Y, Campbell S, Dautzenberg M, Hombergh P van den, Brinkmann H, Szecsenyi J, Falcoff H, Seuntjens L, Kuenzi B, Grol R (2005). Developing a framework of, and quality indicators for, general practice management in Europe. Family Practice.

[B4] European Practice Assessment - EPA. Easy to use and scientifically developed quality management for general practice. http://www.topaseurope.eu/files/EPA-Information-Paper-English-vs11_0.pdf.

[B5] About EquiP. http://www.equip.ch/flx/about_equip/.

[B6] AQUA-Institut GmbH. http://www.aqua-institut.de/stellenangebote.html.

[B7] Obermann K, Müller P (2007). Quality management in private practice. Der Urologe.

[B8] NHG-Praktijkaccreditering^®^, een nieuw stap in de ontwikkeling van het kwaliteitsbeleid in huisartspraktijken. http://npa.artsennet.nl/content/resources/AMGATE_6059_1994_TICH_L962607225/AMGATE_6059_1994_TICH_R176257788546093//.

[B9] General Practice in Australia. http://www.health.gov.au/internet/wcms/Publishing.nsf/Content/pcd-publications-gpinoz2004.

[B10] Boerma WG, Zee J van der, Fleming DM (1997). Service profiles of general practitioners in Europe. European GP Task Profile Study. Br J Gen Pract.

[B11] Kroneman W, Zee J Van der, Groot M (2009). Income development of General Practitioners in eight European countries from 1975 to 2005. BMC Health Services Research.

[B12] Gervas J, Fernandez MP (2006). Western European best practice in primary health care. Eur J Gen Pract.

[B13] Engels Y, Dautzenberg M, Campbell S, Broge B, Boffin N, Marshall M, Elwyn G, Vodopivec-Jamsek V, Gerlach FM, Samuelson M (2006). Testing a European set of indicators for the evaluation of the management of primary care practices. Fam Pract.

[B14] Health system review: Belgium. http://www.euro.who.int/Document/E90059.pdf.

[B15] Kroneman M, Meeus P, Zee J van der, Groot W (2009). The calculation of the Belgian General Practitioner revised. Comment. BMC Health Services Research.

[B16] de Maeseneer J, De Prins L, Heyerick J (1999). Home visits in Belgium: a multivariate analysis. Eur J Gen Pract.

[B17] Remmen R, Seuntjens L, Pestiaux D, Leysen P, Knops K, Lafontaine J-B, Philips H, Lefebvre L, Bruel A Van den, Paulus D (2008). Quality development in general practice in Belgium: status quo or quo vadis?.

[B18] Le carnet de bord "Assurance de Qualité". http://www.maisonmedicale.org.

[B19] Evaluatie van Kwaliteit (EKWA). http://www.domusmedica.be/kwaliteit/ekwa/inleiding-ekwa.html.

[B20] Quality Management in Primary Care, European Practice Assessment. http://www.bertelsmann-stiftung.de/cps/rde/xchg/SID-0A000F0A-2E678D75/bst_engl/hs.xsl/publikationen_2725.htm.

[B21] Annonymous (2008). PROJET DE LOI, portant réforme de l'hôpital et relatif aux patients, à la santé et aux territoires. ASSEMBLÉE NATIONALE, Paris, France.

[B22] Healy J, Sharman E, Lokuge B (2006). Australia: Health system review. Health Systems in Transition.

[B23] A quality framework for Australian general practice: Background paper. http://www.racgp.org.au/Content/NavigationMenu/Advocacy/AqualityframeworkforAustralianGeneralPractice/20060210qualityframe_backgpaper.pdf.

[B24] Hombergh P van den, Künzi B, Szecsenyi J (2007). Workshop European Practice Assessment. Wonca Europe Conference Paris, France.

[B25] Booth BJ, Snowdon T (2007). A quality framework for Australian general practice. Aust Fam Physician.

[B26] Marshall MN, Shekelle PG, McGlynn EA, Campbell S, Brook RH, Roland MO (2003). Can health care quality indicators be transferred between countries?. Qual Saf Health Care.

[B27] Matthys J, De Meyere M, Van Driel ML, de Sutter A (2007). Differences among international pharyngitis guidelines: not just academic. Ann Fam Med.

[B28] The Value of the Divisons Network: An Evaluation of the effect of Divisions of General Practice on Primary Care Performance-No.8. http://melbourneinstitute.com/publications/reports/AScott_8.pdf.

